# Supplementary material for: Tumor copy number alteration burden is a pan-cancer prognostic factor associated with recurrence and death
Source: eLife. 2018 Sep 4;7:e37294. doi: 10.7554/eLife.37294 (PMC6145837; doi:10.7554/eLife.37294)
Supplement: Supplementary file 1. [file elife-37294-supp1.docx]

**Supplementary Table 1. Cohort characteristics**

| **Characteristic** | **Conservative Treatment CNA cohort (n=107)**^a^ | **TAPG1 cohort (n=2333)**^b^ |
| --- | --- | --- |
| Age (years) | 70 (67, 74) | 70 (66, 73) |
| Median follow-up time (years) among survivors | 10.3 (5.0, 14.1) | 14.7 (13.8, 15.8) |
| Patients Underwent Hormonal Treatment | 30 (28%) | 670 (29%) |
| Baseline PSA (ng/ml) | 11.0 (4.0, 28.9) | 13.0 (4.8,30.4) |
| CAPRA score (n=60) | 5 (2, 7) |  |
| Mortality |  |  |
| Death from prostate cancer | 43 | 638 |
| Death from other causes | 43 | 1012 |
| Alive | 21 | 680 |
| Gleason Score at Biopsy |  |  |
| ≤6 | 39 (36%) | 749 (45%) |
| 7 | 26 (24%) | 514 (31%) |
| ≥8 | 42 (39%) | 420 (25%) |
| Clinical Stage |  |  |
| T1 | 20 (33%) | 506 (36%) |
| T2 | 25 (42%) | 612 (44%) |
| T3 | 15 (25%) | 269 (19%) |

Values are displayed as median (IQR) and frequency (percentage).

^a^ n = 60 with available clinical stage

^b^ n = 2170 with available baseline PSA; n = 2330 with available survival status and censoring date; n =1683 with available Gleason and histology; n=1387 with evaluated clinical stage
