# Supplementary material for: Tumor copy number alteration burden is a pan-cancer prognostic factor associated with recurrence and death
Source: eLife. 2018 Sep 4;7:e37294. doi: 10.7554/eLife.37294 (PMC6145837; doi:10.7554/eLife.37294)
Supplement: Supplementary file 2. [file elife-37294-supp2.docx]

**Supplementary Table 2. Distribution of cancer types in IMPACT cohorts**

|  | **Primary tumor cohort** | | | | **Metastatic cohort** | | | |  |
| --- | --- | --- | --- | --- | --- | --- | --- | --- | --- |
|  | **Frequency** | | **Percent** | | **Frequency** | | **Percent** | |  |
| Non-Small Cell Lung | | 1089 | | 16.5 | | 719 | | 14.8 | |
| Breast | | 757 | | 11.5 | | 937 | | 19.3 | |
| Colorectal | | 628 | | 9.5 | | 544 | | 11.2 | |
| Glioma | | 627 | | 9.5 | | 15 | | 0.3 | |
| Prostate | | 394 | | 6.0 | | 335 | | 6.9 | |
| Pancreatic | | 348 | | 5.3 | | 243 | | 5.0 | |
| Bladder | | 329 | | 5.0 | | 105 | | 2.2 | |
| Hepatobiliary | | 262 | | 4.0 | | 102 | | 2.1 | |
| Esophagogastric | | 229 | | 3.5 | | 97 | | 2.0 | |
| Renal Cell Carcinoma | | 220 | | 3.3 | | 144 | | 3.0 | |
| Soft Tissue Sarcoma | | 200 | | 3.0 | | 152 | | 3.1 | |
| Non-Hodgkin Lymphoma | | 175 | | 2.6 | | 12 | | 0.2 | |
| Endometrial | | 166 | | 2.5 | | 127 | | 2.6 | |
| Germ Cell Tumor | | 120 | | 1.8 | | 87 | | 1.8 | |
| Melanoma | | 102 | | 1.5 | | 324 | | 6.7 | |
| Thyroid | | 98 | | 1.5 | | 124 | | 2.5 | |
| Ovarian | | 97 | | 1.5 | | 154 | | 3.2 | |
| Mesothelioma | | 93 | | 1.4 | | 13 | | 0.3 | |
| Head and Neck | | 81 | | 1.2 | | 100 | | 2.1 | |
| Gastrointestinal Stromal Tumor | | 72 | | 1.1 | | 55 | | 1.1 | |
| Bone | | 60 | | 0.9 | | 41 | | 0.8 | |
| Small Cell Lung | | 42 | | 0.6 | | 57 | | 1.2 | |
| Appendiceal | | 41 | | 0.6 | | 45 | | 0.9 | |
| Skin, Non-Melanoma | | 39 | | 0.6 | | 37 | | 0.8 | |
| CNS | | 39 | | 0.6 | | 1 | | <0.1 | |
| Salivary Gland | | 37 | | 0.6 | | 78 | | 1.6 | |
| Embryonal Tumor | | 32 | | 0.5 | | 20 | | 0.4 | |
| Uterine Sarcoma | | 29 | | 0.4 | | 58 | | 1.2 | |
| Small Bowel | | 27 | | 0.4 | | 15 | | 0.3 | |
| Cervical | | 24 | | 0.4 | | 30 | | 0.6 | |
| Ampullary Carcinoma | | 22 | | 0.3 | | 10 | | 0.2 | |
| Gastrointestinal Neuroendocrine Tumor | | 20 | | 0.3 | | 29 | | 0.6 | |
| Anal | | 14 | | 0.2 | | 20 | | 0.4 | |
| Adrenocortical Carcinoma | | 12 | | 0.2 | | 10 | | 0.2 | |
| Nerve Sheath Tumor | | 12 | | 0.2 | | 2 | | <0.1 | |
| Thymic Tumor | | 10 | | 0.2 | | 1 | | <0.1 | |
| Sex Cord Stromal Tumor | | 6 | | 0.1 | | 8 | | 0.2 | |
| Miscellaneous Brain Tumor | | 6 | | 0.1 | | 0 | | 0.0 | |
| Hodgkin Lymphoma | | 6 | | 0.1 | | 0 | | 0.0 | |
| Retinoblastoma | | 6 | | 0.1 | | 0 | | 0.0 | |
| Miscellaneous Neuroepithelial Tumor | | 5 | | 0.1 | | 2 | | <0.1 | |
| Histiocytosis | | 5 | | 0.1 | | 1 | | <0.1 | |
| Vaginal | | 5 | | 0.1 | | 1 | | <0.1 | |
| Wilms Tumor | | 4 | | 0.1 | | 2 | | <0.1 | |
| Penile | | 4 | | 0.1 | | 2 | | <0.1 | |
| Sellar Tumor | | 4 | | 0.1 | | 0 | | 0.0 | |
| Breast Sarcoma | | 3 | | <0.1 | | 2 | | <0.1 | |
| Leukemia | | 3 | | <0.1 | | 1 | | <0.1 | |
| Gestational Trophoblastic Disease | | 3 | | <0.1 | | 0 | | 0.0 | |
| Pheochromocytoma | | 1 | | <0.1 | | 2 | | <0.1 | |
| Multiple Myeloma | | 1 | | <0.1 | | 0 | | 0.0 | |
| Pineal Tumor | | 1 | | <0.1 | | 0 | | 0.0 | |
| Total | | 6610 | | 100 | | 4864 | | 100 | |
