# Supplementary material for: Tumor copy number alteration burden is a pan-cancer prognostic factor associated with recurrence and death
Source: eLife. 2018 Sep 4;7:e37294. doi: 10.7554/eLife.37294 (PMC6145837; doi:10.7554/eLife.37294)
Supplement: Supplementary file 3. — Purity was determined by FACETS (Shen and Seshan, 2016). [file elife-37294-supp3.docx]

**Supplementary Table 3. Association between overall survival and CNA burden after adjustment for purity in IMPACT prostate and pan-cancer cohorts.** Purity was determined by FACETS ([35](#_ENREF_35)).

| ***Model*** | ***Overall Survival*** | | | | | | | | |
| --- | --- | --- | --- | --- | --- | --- | --- | --- | --- |
|  | ***Primary tumors* *^a, c^*** | | | | ***Metastatic tumors* *^b, d^*** | | | | |
|  | **HR** | **95% CI** | | **P** | **HR** | | **95% CI** | | **P** |
| **Prostate Cancer**: Tumor CNA burden, per 5%, adjusted for purity **^a, b^** | 1.04 | | 0.98, 1.11 | NS (0.2) | 1.00 | 0.96, 1.05 | | NS (0.9) | |
| **Pan- Cancer**: Tumor CNA burden, per 5%, adjusted for purity ^c,d^ | 1.02 | | 1.01, 1.03 | 0.002 | 1.01 | 1.00, 1.02 | | NS (0.061) | |

^a^ Prostate primary tumors: patient n=193; event n=28; median follow-up time for survivors 37 (IQR 25,83) months

^b^ Prostate metastatic tumors: patient n=201; event n=77; median follow-up time for survivors 62.5 (IQR 33, 131) months

^c,^ Pan-cancer primary tumors, n=4052

^d^ Pan-cancer metastatic tumors n=3175
