# Supplementary material for: Tumor copy number alteration burden is a pan-cancer prognostic factor associated with recurrence and death
Source: eLife. 2018 Sep 4;7:e37294. doi: 10.7554/eLife.37294 (PMC6145837; doi:10.7554/eLife.37294)
Supplement: Supplementary file 9. [file elife-37294-supp9.docx]

**Supplementary Table 9.** TCGA Cohort statistics: patient exclusion, events, and follow-up

|  | **Initial Cohort Size** | **Unknown CNA Burden** | **Unknown recur-ence status or time** | **Unknown OS status or time to death** | **Immedi- ately censored after treatment** | **Final Cohort**  **Size** | **RF event n** | **Median followup time for RF survivors and IQR (months)** | **OS event n** | **Median followup time for OS survivors and IQR (months)** |
| --- | --- | --- | --- | --- | --- | --- | --- | --- | --- | --- |
| *Prostate* | 330 | 0 | 50 | N/A | 0 | 280 | 32 | 18 (7, 36) | -- | -- |
| *Breast* | 817 | 0 | N/A | 0 | 23 | 794 | 53 | 20 (12, 38) | 100 | 20 (12, 39) |
| *Endometrial* | 547 | 8 | N/A | 2 | 1 | 536 | 105 | 30 (17, 58) | 90 | 31 (18, 58) |
| *Kidney* | 537 | 9 | N/A | 1 | 2 | 525 | 124 | 45 (21, 64) | 175 | 48 (23, 71) |
| *Thyroid* | 507 | 8 | N/A | 1 | 1 | 497 | 46 | 31 (17, 48) | 16 | 31 (18, 50) |
| *Colorectal* | 629 | 13 | N/A | 7 | 22 | 587 | 117 | 22 (13, 34) | 124 | 24 (14, 36) |
